# Comprehensive Analysis of the Expression Characteristics of the Enhancer of the Zeste Homolog 2 Gene in Pan-Cancer

**DOI:** 10.3389/fgene.2021.658241

**Published:** 2021-07-26

**Authors:** Yuanyuan Kang, Ying Zhang, Yan Sun

**Affiliations:** Liaoning Provincial Key Laboratory of Oral Diseases, School and Hospital of Stomatology, China Medical University, Shenyang, China

**Keywords:** EZH2, TCGA, GEO, overall survival, disease-free survival

## Abstract

Although more and more studies have shown that EZH2 was closely related to human cancer, no pan-cancer analysis is available. Therefore, we summarized and analyzed the potential carcinogenic effect of EZH2 for the first time based on TCGA (cancer genome map) datasets. EZH2 is expressed highly in most tumors and there is a significant correlation between the EZH2 expression and the prognosis of patients. We observed the increased phosphorylation levels of T487 in breast cancer, colon cancer, UCEC, and LUAD. The expression of EZH2 was associated with the CD8+, tregs, macrophage, and cancer-associated fibroblast infiltration in some tumors. In addition, the cell cycle and cellular biology were involved in the functional mechanisms of EZH2. Our study summarized and analyzed the carcinogenic effect of EZH2 in different tumors comprehensively and provided a theoretical basis for targeting EZH2 therapy.

## Introduction

Due to the complexity of tumorigenesis, more and more attention has been paid to the pan-cancer analysis of the same gene, which is not only helpful to discover the common phenotypic characteristics of tumors but also helps us to understand the causes of key molecular events and their own internal regulatory mechanisms deeply.

Enhancer of zeste homolog 2 (EZH2) is a chromatin-modifying protein, which is the core catalytic subunit of polycomb repressive complex 2 (PRC2). Its expression or functional status can affect the trimethylation of histone 3 on lysine 27 (H3K27me3) by PRC2, thus inducing the chromatin conformation changes of downstream genes and causing transcriptional inhibition or activation (Comet et al., 2016). In addition, it also participates in the regulation of multiple signaling pathways and biological processes independent of PRC2. Previous studies have shown that EZH2 was closely related to the occurrence and development of tumors. High expression of EZH2 is found in breast cancer (Jang et al., 2016), prostate cancer (Wu et al., 2016), pancreatic cancer (Kuroki et al., 2014), gastric cancer (Guo et al., 2014), and other malignant tumors. The high expression of EZH2 maintains the high proliferation and metastasis of tumors and was related to the malignancy and poor prognosis of tumors closely. However, there is still no pan-cancer evidence on the relationship between EZH2 and various tumor types based on big clinical data.

In our study, we reveal the relationship between the gene expression, mutation, protein phosphorylation, DNA methylation, immune infiltration, and prognostic potential of the EZH2 gene in pan-cancer by analyzing the data from the TCGA project, clarify the important role of the EZH2 gene in tumorigenesis and the possible regulatory mechanism. This study is helpful to understand the pathogeny of abnormal expression of EZH2 and its underlying regulatory mechanism and provide a basis for targeted therapy of EZH2 related tumors.

## Materials and Methods

### Gene Expression Analysis

The tumor immune estimation resource, version 2 (TIMER2) web server^1^ (Tang et al., 2019) was used to explore the differential EZH2 expression between tumor and normal tissues in TCGA tumors. Enter the gene symbol (EZH2) in the “Exploration” field. The “Submit” button was clicked to generate the expression profile of the EZH2 in all tumor and normal tissues.

For certain tumors without the control [e.g., adrenocortical carcinoma (ACC), lymphoid neoplasm diffuse large B-cell lymphoma (DLBC), acute myeloid leukemia (LAML), brain lower grade glioma (LGG), ovarian serous cystadenocarcinoma (OV), sarcoma (SARC), skin cutaneous melanoma (SKCM), thymoma (THYM), testicular germ cell tumors (TGCT), and uterine carcinosarcoma (UCS)], the gene expression profiling interactive analysis, version 2 (GEPIA2) web server was used. Enter the gene symbol (EZH2) in the “Expression DIY” field of “Box Plots” module, the “Plot” button was clicked to generate the expression profile, with the settings of log_2_FC (Fold change) cutoff = 1, *P*-value cutoff = 0.01, and “Match TCGA normal and GTEx data.”

Additionally, the GEPIA2 web server was used to explore the correlation between EZH2 expression and tumor pathological stages. Under the “Stage Plot” module of GEPIA2, the results of the EZH2 expression in different stages (stage I, II, III, and IV) of tumors were presented in the form of violin plots.

The UALCAN^2^ is an interactive web resource for analyzing the TCGA database, allowed us to conduct protein expression analysis of the clinical proteomic tumor analysis consortium (CPTAC) dataset (Chen et al., 2019). Herein, the total EZH2 protein or phosphoprotein (with phosphorylation at the T487) were both explored between the primary tumor and the control.

### Reverse-Transcription Polymerase Chain Reaction (RT-qPCR)

Total RNA was extracted using TRIzol (TaKaRa, Dalian, China). The extracted RNA was used to generate cDNA using the PrimeScript RT cDNA Synthesis kit (TaKaRa). cDNAs were quantified by quantitative real-time PCR using SYBR Premix Ex Taq (TaKaRa) on a Mx3000P instrument (Agilent Stratagene). Gene expression was normalized to GAPDH expression, which was used as an internal control, and the relative expression level was calculated using the 2^–ΔΔCt^ method, *P* < 0.05 indicated statistical significance.

### Western Blotting

Proteins were extracted and the concentrations were determined by the bicinchoninic acid method. Samples containing 50 μg of total proteins were separated by SDS-PAGE on a 10% gel and then transferred to polyvinylidene difluoride (PVDF) membranes. After blocking with 5% non-fat milk, membranes were immunoblotted with antibodies overnight, followed by secondary antibodies. The proteins were visualized using Thermo Pierce ECL (Thermo Fisher Scientific, Waltham, MA, United States).

### Survival Prognosis Analysis

The GEPIA2 web server was used to analyze the survival prognosis. In the “Survival Map” module, enter the “EZH2,” the results of the overall survival (OS) and disease-free survival (DFS) significance map data were then presented under the settings of Group cutoff = median. The results in the form of survival plots were also presented through the “Survival Analysis” module of GEPIA2 and Kaplan–Meier plots with log-rank *P*-value were generated.

### Genetic Alteration Analysis

The cBioPortal web server^3^ (Cerami et al., 2012; Gao et al., 2013) was used to explore genetic alteration. In the “Quick select” section, the “TCGA Pan Cancer Atlas Studies,” was chosen and the “Query By Gene” button was clicked. The command to enter “EZH2” for the gene name, and the results were presented through the “Cancer Types Summary” module in the form of bar plots and the alteration frequency, mutation, and copy number alteration in all TCGA tumors were observed. The mutation site information of EZH2 was displayed in the protein structure diagram of the “Mutation” module.

Additionally, the OS, disease-free, progression-free, and DFS with EZH2 genetic alteration were detected after using the “Comparison” module. Kaplan–Meier plots with a log-rank *P*-value were generated.

### Immune Infiltration Analysis

The IMER2 web server was used to explore the correlation between EZH2 expression and immune infiltration. We entered “EZH2” for gene name, the immune cells were selected, and the results were presented through the form of a heatmap and scatter plots. The immune infiltration estimations were conduct by using EPIC, MCPCOUNTER, XCELL, and TIDE algorithms. The Spearman’s rank correlation test was used.

### EZH2-Related Gene Enrichment Analysis

The STRING website^4^ was used to screen the proteins which bind to EZH2. Entering the “EZH2” for protein name, we obtain the available experimentally determined EZH2-binding proteins with the settings of network type (“full network”), meaning of network edges (“evidence”), active interaction sources (“experiments”), minimum required interaction score [“Low confidence (0.150)”], and the max number of interactors to show (“no more than 50 interactors” in 1st shell).

The “Similar Gene Detection” module of GEPIA2 was used to screen the top 100 EZH2-correlated targeting genes. At the same time, the results were presented in the form of a dot plot after the “correlation analysis” module of GEPIA2 was used to perform a pairwise gene Pearson correlation between EZH2 and the selected genes. Moreover, enter the “EZH2” in the “Gene_Corr” module, the results of the heatmap data were then presented. The Spearman’s rank correlation test was used.

The Jvenn website^5^, an interactive Venn diagram viewer (Bardou et al., 2014) was used to take an intersection analysis between the 50 EZH2-binding protein and top 100 interacted genes. In addition, the DAVID website^6^ and the “ggplot2”^7^ R packages were used to analyze the Kyoto Encyclopedia of Genes and Genomes (KEGG) pathway. The results were visualized as bubble chart. Finally, we used the “clusterProfiler” R package and R language software^8^ to analyze the gene ontology (GO) enrichment. The results were visualized as bar plots.

## Results

### Gene Expression

**FIGURE 1:**
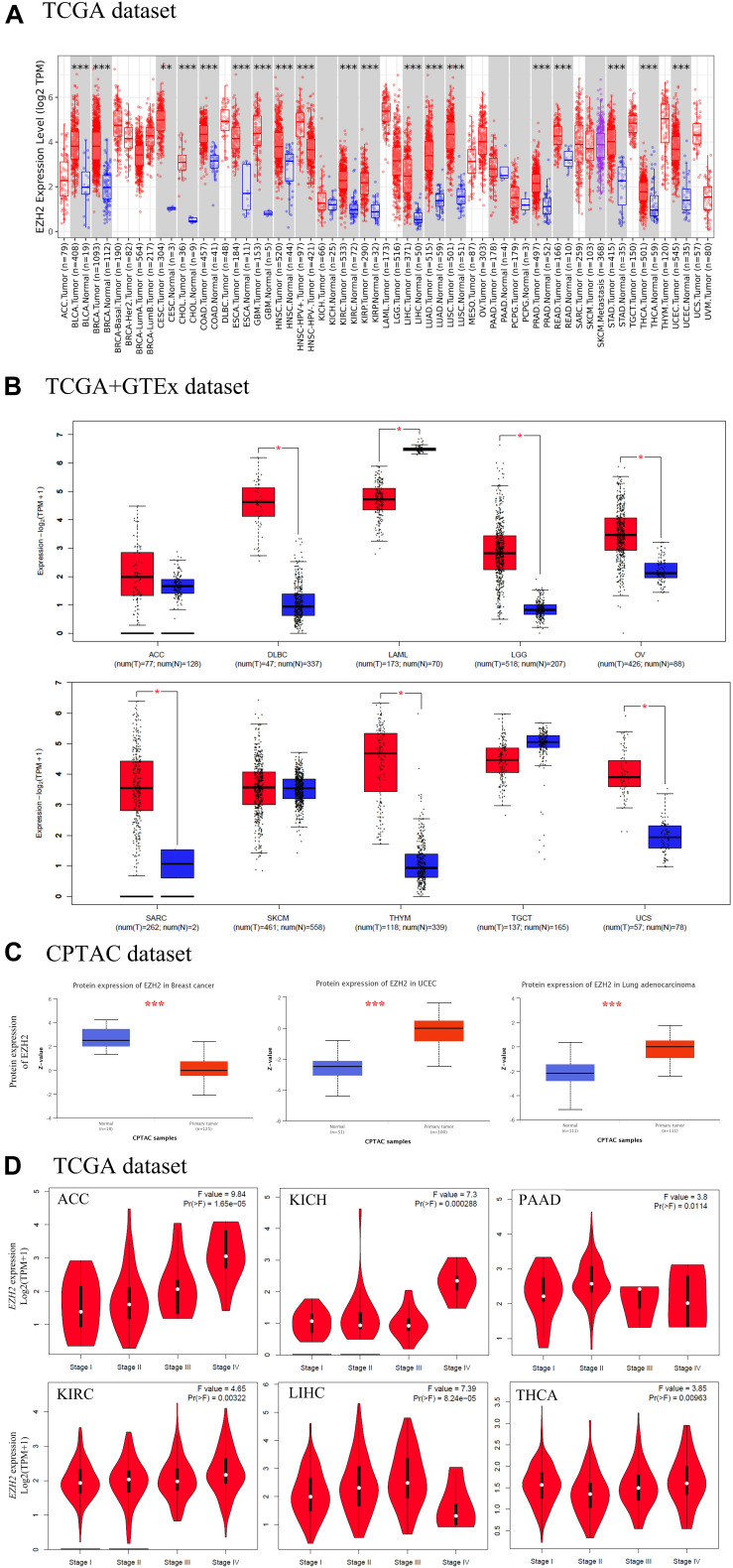
The expression level of EZH2 gene in different tumors. **(A)** The expression status of the EZH2 gene in different cancers through TIMER2. **(B)** For the type of ACC, DLBC, LAML, LGG, OV, SARC, SKCM, THYM, TGCT, and UCS in the TCGA project, the corresponding normal tissues of the GTEx database were used as controls. **(C)** The expression level of EZH2 total protein between normal tissue and primary tissue of breast cancer, UCEC and LUAD were analyzed based on the CPTAC dataset. **(D)** The correlation between EZH2 expression and the pathological stages of cancers (ACC, KICH, PAAD, KIRC, LIHC, and THCA) were analyzed based on the TCGA data. *P < 0.05; **P < 0.01; ***P < 0.001.

We found that EZH2 expression in tumor tissues of bladder urothelial carcinoma (BLCA), breast invasive carcinoma (BRCA), cholangiocarcinoma (CHOL), colon adenocarcinoma (COAD), esophageal carcinoma (ESCA), glioblastoma multiforme (GBM), head and neck squamous cell carcinoma (HNSC), kidney renal clear cell carcinoma (KIRC), kidney renal papillary cell carcinoma (KIRP), liver hepatocellular carcinoma (LIHC), lung adenocarcinoma (LUAD), lung squamous cell carcinoma (LUSC), prostate adenocarcinoma (PRAD), rectum adenocarcinoma (READ), stomach adenocarcinoma (STAD), thyroid carcinoma (THCA), uterine corpus endometrial carcinoma (UCEC) (*P* < 0.001), and cervical squamous cell carcinoma and endocervical adenocarcinoma (CESC) (*P* < 0.01) is higher than the corresponding control tissues in TCGA dataset (Figure 1A).

The normal tissues of the GTEx dataset were used as controls. We found that EZH2 expression in tumor tissues of DLBC, LGG, OV, SARC, THYM, and UCS was higher than the corresponding control tissues (*P* < 0.05); the expression of EZH2 in tumor tissues of LAML was lower than the corresponding control tissues (*P* < 0.05). However, we did not obtain a significant difference for other tumors, such as ACC, SKCM, and TGCT (Figure 1B).

The CPTAC dataset was used to analyze the EZH2 total protein in different malignant tumors of TCGA. We found that the EZH2 protein in tumor tissues of UCEC and LUAD were higher than the normal tissues, and the result of the breast cancer was the opposite (Figure 1C, *P* < 0.001).

The GEPIA2 tool was also used to observe the EZH2 expression in different tumor stages. We found that there exists a correlation between EZH2 expression and the pathological stages of cancers, including ACC, KICH, PAAD, KIRC, LIHC, and THCA (Figure 1D, all *P* < 0.05).

**FIGURE 2:**
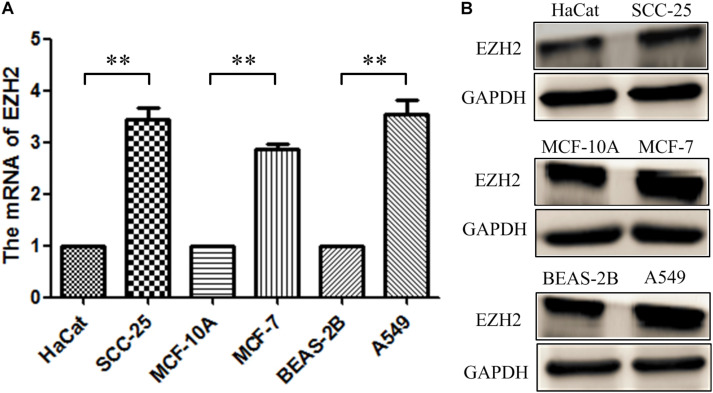
The expression of EZH2 in three tumor cells. **(A)** RT-qPCR. **(B)** Western blotting. **P < 0.01.

RT-qPCR and Western blot were conducted to measure the EZH2 expression in three tumor cells. As shown in Figure 2, the relative expression levels of EZH2 mRNA and protein in tumor cells were up-regulated, which were similar to those observed in the bioinformatics analysis.

### Survival Analysis

**FIGURE 3:**
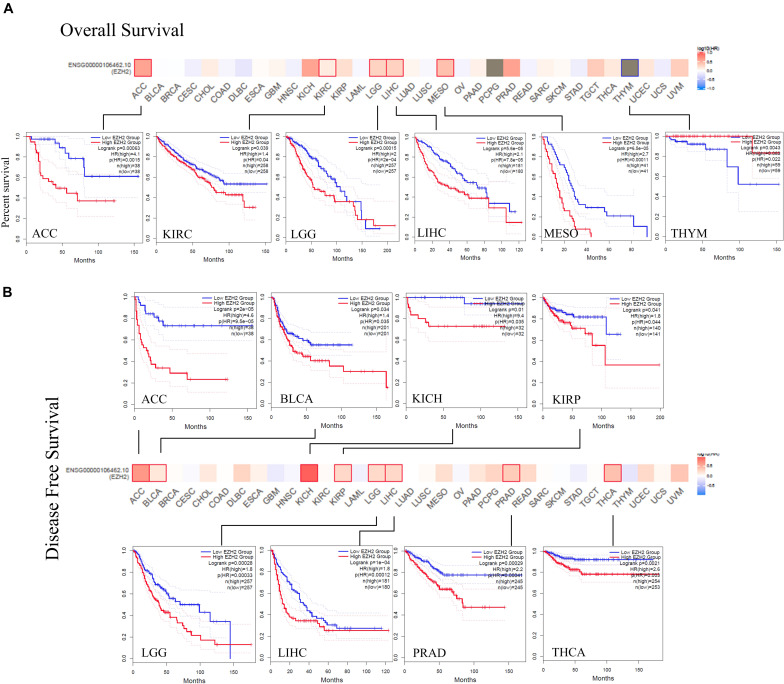
Correlation between EZH2 gene expression and survival prognosis of cancers based on the TCGA data. The overall survival **(A)** and disease-free survival **(B)** analyses of different tumors by EZH2 gene expression were perform in the form of a survival map and Kaplan–Meier curves.

The prognostic role of EZH2 in tumors was present. According to the expression levels of EZH2, the cancer cases were divided into high-expression and low-expression groups. We found that cases with higher EZH2 expression had poor OS for ACC (*P* = 0.0015), KIRC (*P* = 0.04), LGG (*P* = 2e-04), LIHC (*P* = 7.8e-05), and MESO (*P* = 0.00011), but lower EZH2 expression was related to poor OS prognosis for THYM (*P* = 0.022) (Figure 3A). DFS analysis data showed a correlation between high EZH2 expression and poor prognosis for cases of ACC (*P* = 9.5e-05), BLCA (*P* = 0.035), KICH (*P* = 0.035), KIRP (*P* = 0.044), LGG (*P* = 0.00033), LIHC (*P* = 0.00012), PRAD (*P* = 0.00041), and THCA (*P* = 0.003) (Figure 3B).

### Genetic Alteration Analysis

**FIGURE 4:**
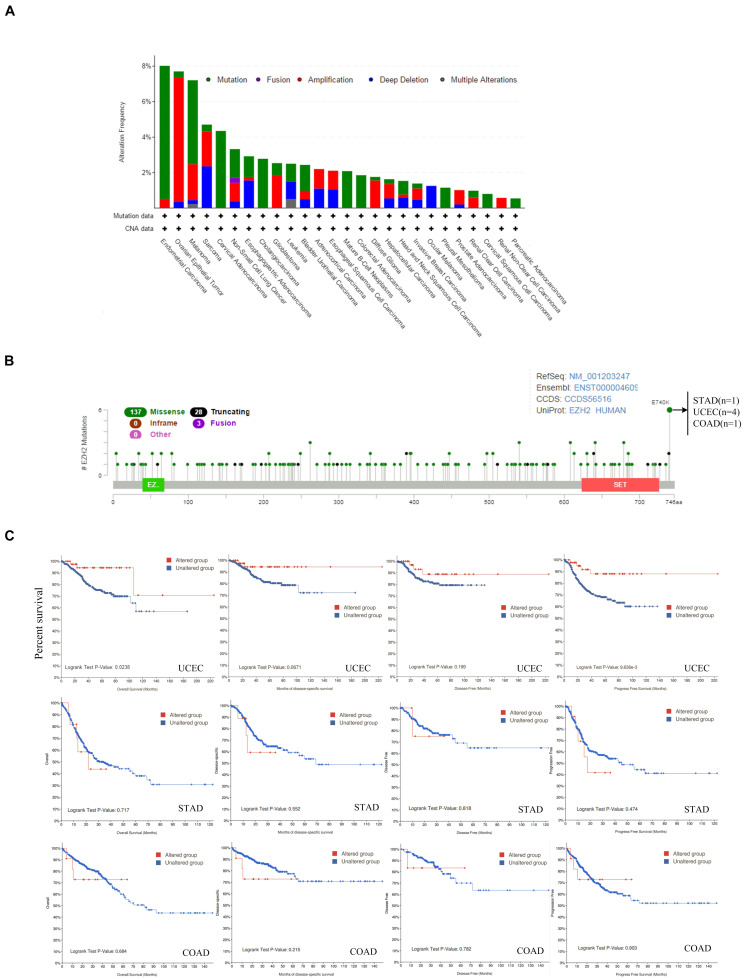
Mutation feature of EZH2 in different tumors of TCGA. The alteration frequency with mutation type **(A)** and mutation site **(B)** are displayed. **(C)** The potential correlation between mutation status and overall, disease-specific, disease-free, and progression-free survival of UCEC, STAD, and COAD was investigated.

The genetic alteration status of EZH2 in different tumors of the TCGA cohorts was observed. We found that the patients with endometrial carcinoma had the highest alteration frequency of EZH2 (=8%) with “mutation” as the primary type. The patients with ovarian epithelial tumors had the second-highest alteration frequency of EZH2 (<8%) with the “amplification” type as the primary type. “Mutation” as the only form of variation existed in all cases of cervical adenocarcinoma, cholangiocarcinoma, mature B-cell neoplasm, colorectal adenocarcinoma, pleural mesothelioma, cervical squamous cell carcinoma, and pancreatic adenocarcinoma. It is worth noting that all cases of ocular melanoma cases with genetic alteration (<2%) had copy number deletion of EZH2. The remaining tumors contained more than two variations (Figure 4A). Figure 4B showed the sites, types, and case number of the EZH2 genetic alteration. We found that the main type of genetic change of EZH2 was missense mutation. The alteration of E740K, which was detected in four cases of UCEC, one case of STAD (Stomach adenocarcinoma), and one case of COAD.

Additionally, the potential relationship between genetic alteration of EZH2 and the clinical survival prognosis with UCEC, STAD, and COAD were explored. We found that compared with the unaltered EZH2 group, the UCEC cases with the altered EZH2 group showed better prognosis in terms of OS (*P* = 0.0238) and PFS (progression-free survival) (*P* = 9.636e-3) but not DSF (*P* = 0.0671) and DF (disease-free) (*P* = 0.199); in STAD and COAD, however, we found that there was no significant difference (Figure 4C).

### Protein Phosphorylation and DNA Methylation Analysis

**FIGURE 5:**
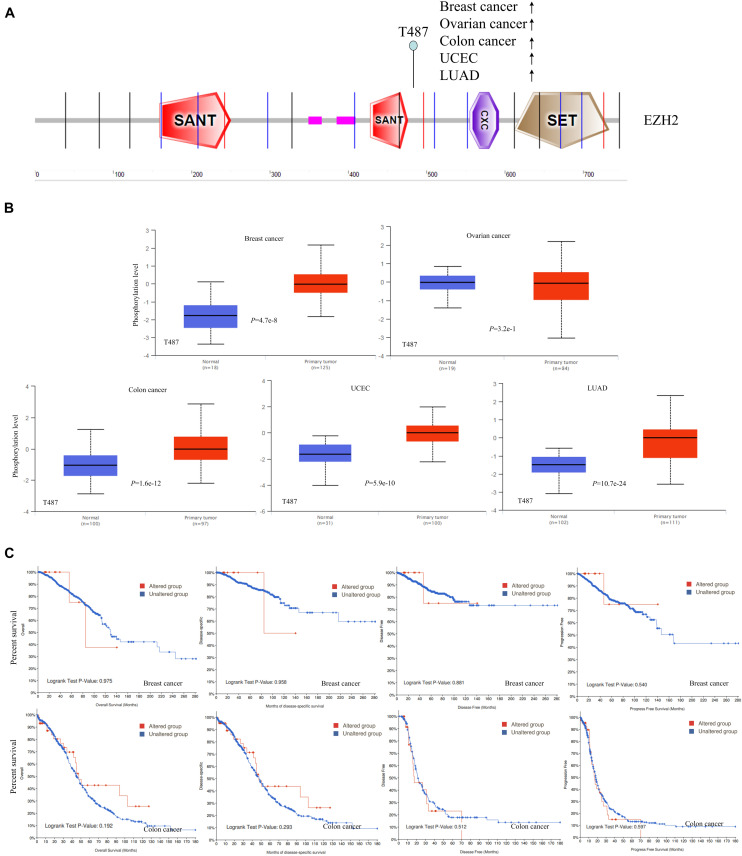
Phosphorylation analysis of EZH2 protein in different tumors. **(A)** The phosphoprotein sites (T487) are displayed in the schematic diagram of the EZH2 protein. **(B)** The box plots for different cancers, including breast cancer, ovarian cancer, colon cancer, UCEC, and LUAD. **(C)** The potential association between phosphorylation level of EZH2 and the clinical survival prognosis of cases with breast cancer and colon cancer.

The differences in EZH2 phosphorylation levels in different malignant tumors were also compared by using the CPTAC dataset. Figure 5 indicated that the T487 locus exhibits a higher phosphorylation level for breast cancer (*P* = 4.7e-8), colon cancer (*P* = 1.6e-12), UCEC (*P* = 5.9e-10), and LUAD (*P* = 10.7e-24) with normal tissues (Figure 5A), followed by no significant change of phosphorylation level of the T487 locus for ovarian cancer (*P* = 3.2e-1) (Figure 5B). Additionally, the potential association between the phosphorylation level of EZH2 and the clinical survival prognosis of cases with breast cancer and colon cancer were explored. We found that there was no significant difference (Figure 5C).

**FIGURE 6:**
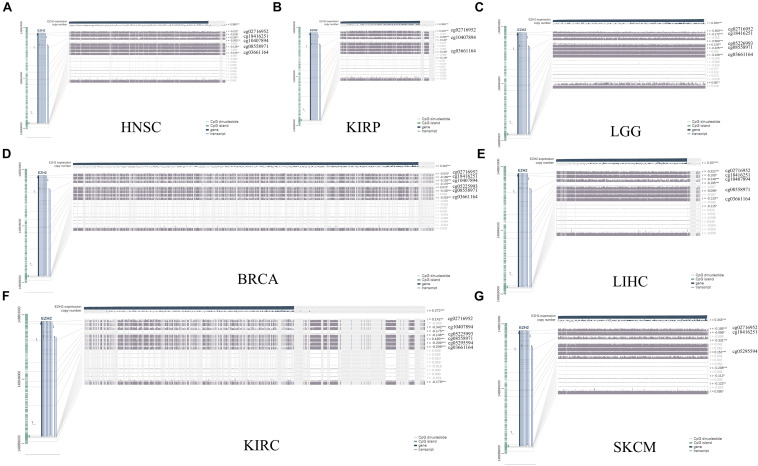
DNA methylation analysis of EZH2 in **(A)** HNSC, **(B)** KIRP, **(C)** LGG, **(D)** BRCA, **(E)** LIHC, **(F)** KIRC, and **(G)** SKCM. *P < 0.05, **P < 0.01, ***P < 0.001.

The MEXPRESS approach was used to investigate the potential association between EZH2 DNA methylation and the pathogenesis of different tumors in the TCGA project. We observed a significant negative correlation of EZH2 DNA methylation and gene expression at multiple probes of the non-promoter region, such as cg02716952 and cg03661164, as shown in Figure 6.

### Immune Infiltration Analysis

**FIGURE 7:**
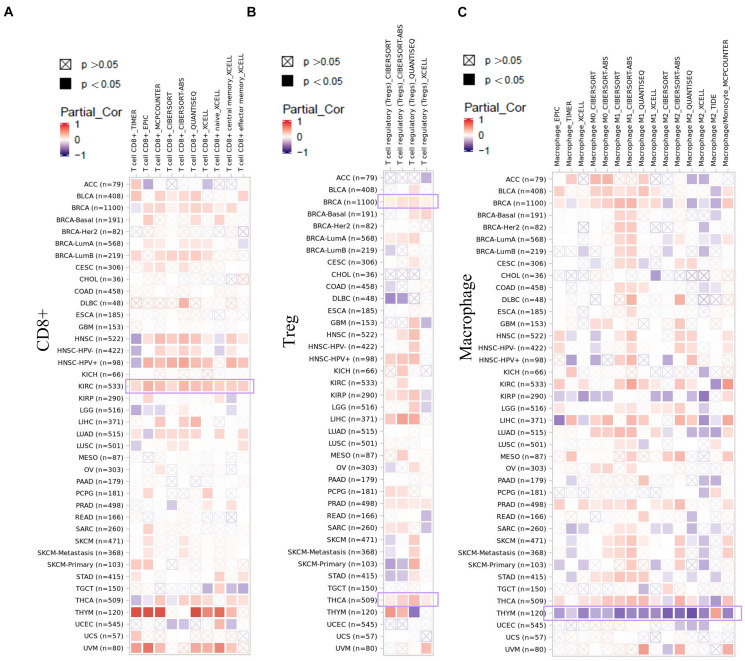
Correlation analysis between EZH2 expression and immune infiltration of **(A)** CD8+, **(B)** tregs, and **(C)** macrophages.

As an important part of the tumor microenvironment, tumor-infiltrating immune cells are closely related to the occurrence, development, and metastasis of tumors (Fridman et al., 2011; Steven and Seliger, 2018). Herein, in this study, the potential relationship between the infiltration level of different immune cells and EZH2 gene expression in diverse cancer types of TCGA was investigated. We observed a statistically positive correlation between the immune infiltration of CD8+ cells and EZH2 expression in the tumors of KIRC. EZH2 expression was positively associated with the infiltration of tregs in BRCA and THCA. There was a positive correlation between EZH2 and the infiltration of macrophages in THYM (Figure 7).

**FIGURE 8:**
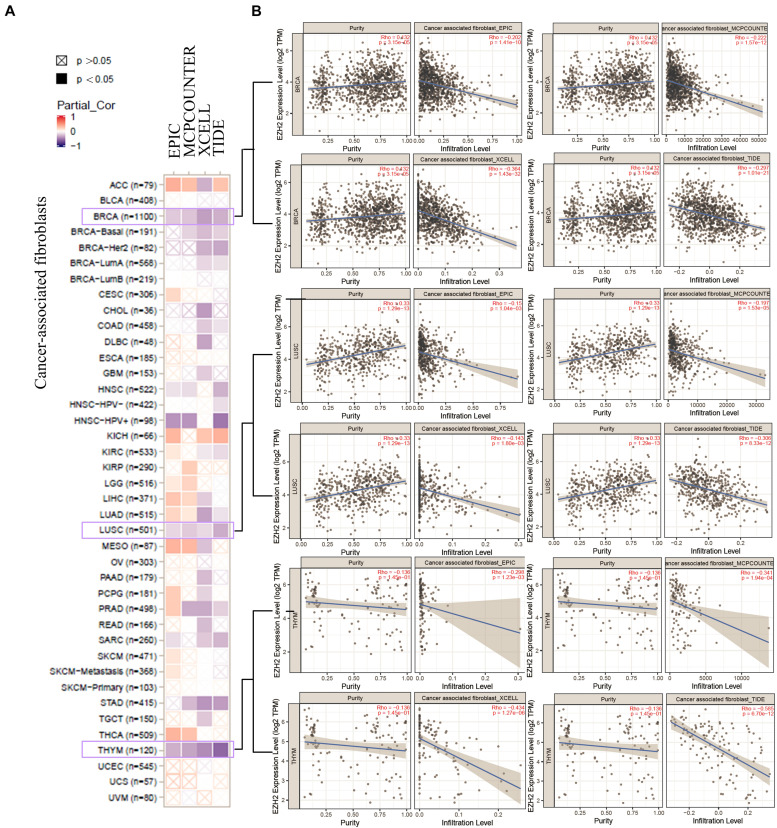
Correlation analysis between EZH2 expression and cancer-associated fibroblasts. **(A)** Four algorithms (EPIC, MCPCOUNTER, XCELL, and TIDE) were used to explore the correlation between EZH2 expression and the infiltration level of cancer-associated fibroblasts as the form of a survival map. **(B)** Scatter plots were present for BRCA, LUSC, and THYM patients.

**FIGURE 9:**
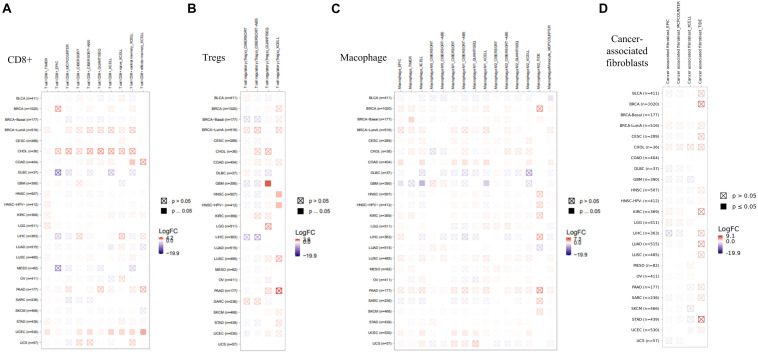
Correlation analysis between EZH2 expression and immune infiltration of **(A)** CD8+, **(B)** Tregs, **(C)** Macophage, **(D)** Cancer-associated fibroblasts.

We also found a statistically positive correlation between EZH2 expression and cancer-associated fibroblasts for the TCGA tumors of BRCA, LUSC, and THYM (Figure 8A). The scatter plot data of the three tumors produced using four algorithms (EPIC, MCPCOUNTER, XCELL, and TIDE) are presented in Figure 8B. For example, the EZH2 expression level in BRCA is positively correlated with the infiltration level of cancer-associated fibroblasts (Rho = −0.202, *P* = 1.41e-10) based on the EPIC algorithm. Additionally, the potential relationship between EZH2 mutation expression and immune infiltration was also investigated. There was a positive correlation between EZH2 mutation and the CD8+ macrophage in UCEC (Figure 9).

### Enrichment Analysis

**FIGURE 10:**
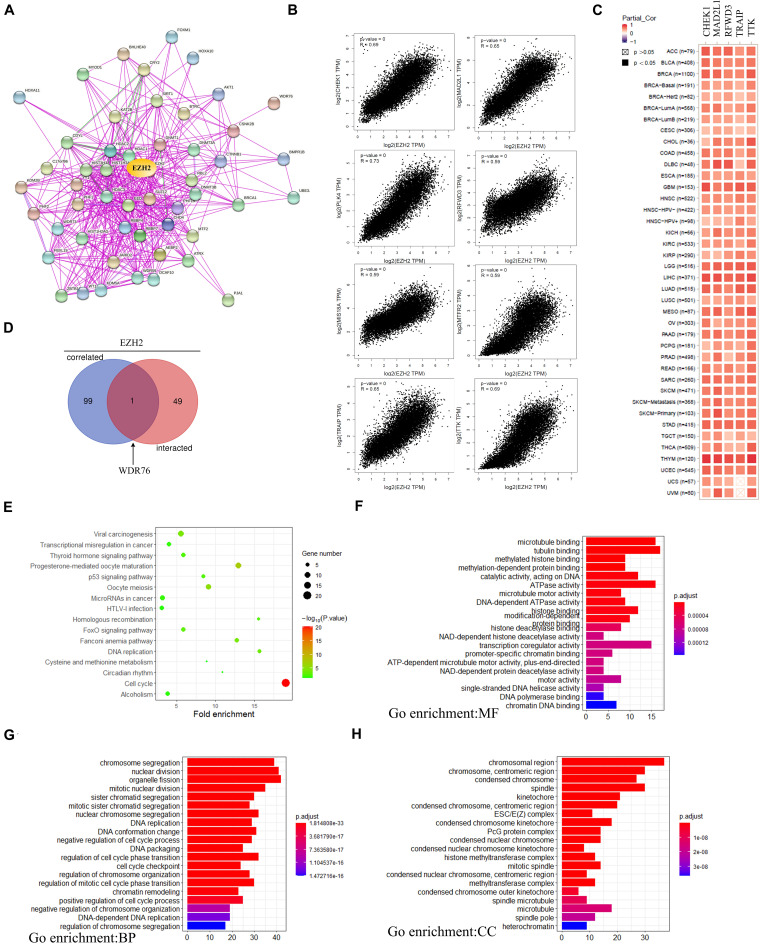
EZH2-related gene enrichment analysis. **(A)** The EZH2-binding proteins using the STRING tool. **(B)** The top 100 EZH2-correlated genes in TCGA projects and analyzed the correlation between EZH2 and selected targeting genes. **(C)** The corresponding heatmap data in the detailed cancer types are displayed. **(D)** An intersection analysis of the EZH2-binding and correlated genes was performed. **(E)** KEGG pathway analysis was performed. **(F–H)** The GO pathway analysis was performed.

In order to further study the mechanism of the EZH2 gene in tumorigenesis, we tried to screen EZH2 binding protein and EZH2 expression-related genes for a series of pathway enrichment analyses. As Figure 10A shown, we obtained a total of 50 EZH2-binding proteins, which were supported by using the STRING tool. The GEPIA2 tool was also used to obtain the top 100 genes that correlated with EZH2 expression. We found that the EZH2 expression level was correlated with that of CHEK1 (*R* = 0.69), MAD2L1 (*R* = 0.65), PLK4 (*R* = 0.73), RFWD3 (*R* = 0.59), MIS18A (*R* = 0.59), MTFR2 (*R* = 0.59), TRAIP (*R* = 0.65), and TTK (*R* = 0.69) genes positively (Figure 10B). There exist a positive correlation between EZH2 and the five genes (CHEK1, MAD2L1, RFWD3, TRAIP, and TTK) in the majority of detailed cancer types as shown in the form of heatmap data (Figure 10C).

One gene (WDR76) was screened by crossing the top 100 related gene with 50 binding protein interacting genes by using the Venn website (Figure 10D). Next, KEGG and GO enrichment analyses were analyzed to predict the functions of EZH2. KEGG analysis can define the pathways related to the functions of EZH2 alterations and the frequently altered neighbor genes. We found that “Cell cycle” and “Viral carcinogenesis” might be involved in the effect of EZH2 on tumor pathogenesis (Figure 10E). Go enrichment analysis predicted the function of target host genes from three aspects: biological process (BP), cell composition (CC), and molecular function (MF). We found that most of these genes are linked to the pathways or cellular biologies, such as tubulin binding, organelle fission and chromosomal region, and others (Figure 10F–H).

## Discussion

Polycomb group (PcG) protein is a group of conserved transcription repressor which participates in the regulation of gene transcription. The abnormal expression of PcG was related to the occurrence and development of tumor closely, and EZH2 is the core component. The human EZH2 gene was located on chromosome 7q35-7q36 (Hillier et al., 2003), which contains 20 exons and encodes 746 amino acids. By catalyzing the trimethylation of histone H3K27, EZH2 mainly causes gene silencing to play the regulatory role of epigenetics. EZH2 can promote cancer development and metastasis (Simon and Lange, 2008; Chang and Hung, 2012; Lu et al., 2016). EZH2 can regulate many cellular processes, such as migration, cell cycle, proliferation, DNA repair, apoptosis, and senescence to facilitate cell survival or promote the malignant transformation of cells (Li et al., 2017; Huang et al., 2018; Ito et al., 2018). However, up to now, we did not find any publications on a pan-cancer analysis of EZH2 from the perspective of the overall tumor. Therefore, based on the data of TCGA databases, we conducted a comprehensive detection of the EZH2 gene in 33 different tumors from the aspects of gene expression, mutation, protein phosphorylation, and so on.

Many studies showed that EZH2 was upregulated in various solid malignancies including lung, hepatocellular, colorectal, breast, and pancreatic cancer, etc. (Gan et al., 2018). In our study, we first investigated the EZH2 expression in various tumors according to the TCGA. The result showed that expression of EZH2 in most tumor tissues was expressed highly except for the LAML. In recent years, the tumor-suppressive roles of EZH2 were also identified. Suppression of EZH2 promotes cancer progression in some cancer types. Basheer et al. (2019) found that in mouse models of LAML, deletion of EZH2 before retroviral transduction with oncogenic MLL-AF9 or AML1-ETO9a fusion genes accelerated disease and shortened survival, indicating that EZH2 functions as a tumor suppressor, which was the same as the result of our findings.

Several studies have shown that EZH2 was closely related to the survival of cancer patients. Our finding showed that cases with higher expression EZH2 had a poor prognosis for cancers of ACC, LGG, and LIHC. To explain the discrepancy in that EZH2 is highly expressed in the THYM, by contrast, this high expression status is correlated with a good prognosis of cases. First, it should be noted that the number of cases in the high-expression or low-expression EZH2 groups is no more than 100, perhaps a larger sample size is necessary to verify the above conclusion. Second, more in-depth molecular experimental evidence is needed to determine whether the high expression of EZH2 plays an important role in the initiation of the above tumors or whether it is only the result of resisting tumor changes in normal tissues.

Through our analysis of reproductive system tumors, we predict that there exist special regulatory mechanisms. Yi et al. (2017) found that EZH2 inhibits TIMP2 expression via H3K27me3 and DNA methylation by which mean it relieves MMP repression and facilitates migration and invasion of ovarian cancer cells, but we failed to observe a positive correlation between EZH2 expression and the survival prognosis of patients with OV in the TCGA cohort. Similarly, although EZH2 was participated in the EZH2/Forkhead box M1 (FoxM1) complex and induces MMP expression and invasion in triple-negative breast cancer (Mahara et al., 2016), we did not find the correlation between EZH2 and tumor prognosis based on the TGCA cohort.

The gene variation types include the following: mutation, fusion, amplification, deep deletion, and multiple alterations. Our finding found that the “mutation” and “fusion” were the two main forms of EZH2 variation in all tumors. “Mutation” was the only form of variation that existed in all cases of cervical adenocarcinoma, cholangiocarcinoma, mature B-cell neoplasm, colorectal adenocarcinoma, pleural mesothelioma, cervical squamous cell carcinoma, and pancreatic adenocarcinoma. It is worth noting that all cases of ocular melanoma cases with genetic alteration had copy number deletion of EZH2.

Phosphorylation is an essential regulatory mechanism in several proteins (Beausoleil et al., 2004). Phosphorylation, which usually occurs on serine (S), threonine (T), and tyrosine (T) residues of substrates, induces a conformational change in many proteins, causing them to be activated or suppressed and consequently creating different biological functions (Johnson and Hunter, 2005). As early as 2005, Cha et al. (2005) showed phosphorylation of EZH2 at S21 (pS21-EZH2) by PI3K/AKT signaling in breast cancer cells. The findings of our study indicated a high expression level of EZH2 total protein and phosphorylation level at the T487 locus in the primary tumors compared with normal controls. Some research reported that the phosphorylation of T487 is catalyzed by CDK1, which is essential for the modulation of the G1/S phase transition during the cell cycle. Therefore, additional experiments are required to further evaluate the potential role of EZH2 phosphorylation at the T487 site and the related cell cycle regulation in tumorigenesis.

Cancer-associated fibroblasts (CAFs) play an important role in promoting the growth and progression of tumors, which has become the subject of extensive research in the field of tumors. Early studies have shown that CAFs promote tumor growth by inducing angiogenesis (Yang et al., 2017) and invasion (Liao et al., 2009). Huang et al. (2019) showed that CAFs promote angiogenesis of hepatocellular carcinoma via the vascular endothelial growth factor-mediated EZH2/vasohibin 1 pathway and may be a potentially useful therapeutic target for hepatocellular carcinoma. Our finding found that there exists a statistical positive correlation between EZH2 expression and CAF for certain tumors, such as BRCA, LUSC, and THYM.

In summary, in this study, we explored the expression of EZH2 mutation, the gene variation of EZH2, and the relationship between the prognosis potential of the EZH2 gene and the whole tumor by using the public database. This study provides detailed data for further understanding of the relationship between EZH2 phenotype and different tumors, provides a theoretical basis for further understanding of the causes of abnormal expression of EZH2 and its internal regulatory mechanism, and provides new research clues for the development of targeted therapy of related tumors targeting EZH2.

## Data Availability Statement

The original contributions presented in the study are included in the article/supplementary material, further inquiries can be directed to the corresponding author.

## Author Contributions

YK and YZ conceived and designed the study. YS analyzed the data. YK wrote the manuscript. All authors contributed to the article and approved the submitted version.

## Conflict of Interest

The authors declare that the research was conducted in the absence of any commercial or financial relationships that could be construed as a potential conflict of interest.

## Publisher’s Note

All claims expressed in this article are solely those of the authors and do not necessarily represent those of their affiliated organizations, or those of the publisher, the editors and the reviewers. Any product that may be evaluated in this article, or claim that may be made by its manufacturer, is not guaranteed or endorsed by the publisher.

## Footnotes

1 http://timer.cistrome.org/

2 http://ualcan.path.uab.edu/index.html

3 https://www.cbioportal.org/

4 https://string-db.org/

5 http://bioinformatics.psb.ugent.be/webtools/Venn/

6 https://david.ncifcrf.gov/

7 https://cran.r-project.org/web/packages/ggplot2/index.html

8 https://www.r-project.org/
